# Secretory pathway of cellulase: a mini-review

**DOI:** 10.1186/1754-6834-6-177

**Published:** 2013-12-02

**Authors:** Shaomin Yan, Guang Wu

**Affiliations:** 1 State Key Laboratory of Non-food Biomass Enzyme Technology, National Engineering Research Center for Non-food Biorefinery, Guangxi Key Laboratory of Biorefinery, Guangxi Academy of Sciences, 98 Daling Road, Nanning, Guangxi 530007, China; 2 DreamSciTech, Apt 207, Zhencaili 26, Zhujiang Road, Hexi District, Tianjin, 300222, China

**Keywords:** Cellulase, Secretory pathway, Subcellular location, Secretory mechanism, UniProtKB

## Abstract

Cellulase plays an important role in modern industry and holds great potential in biofuel production. Many different types of organisms produce cellulase, which go through secretory pathways to reach the extracellular space, where enzymatic reactions take place. Secretory pathways in various cells have been the focus of many research fields; however, there are few studies on secretory pathways of cellulases in the literature. It is therefore necessary and important to review the current knowledge on the secretory pathways of cellulases. In this mini-review, we address the subcellular locations of cellulases in different organisms, discuss the secretory pathways of cellulases in different organisms, and examine the secretory mechanisms of cellulases. These sections start with a description of general secreted proteins, advance to the situation of cellulases, and end with the knowledge of cellulases, as documented in UniProt Knowledgebase (UniProtKB). Finally, gaps in existing knowledge are highlighted, which may shed light on future studies for biofuel engineering.

## Introduction

Cellulase is a word by combining cellul(ose) with -ase, and appeared around 1900 to 1905 [1], whereas the word cellulose appeared between 1745 and 1755 [2]. Cellulase belongs to EC 3.2.1.4 and catalyzes the endohydrolysis of 1,4-beta-D-glucosidic linkages in cellulose, lichenin, and cereal beta-D-glucans [3]. In a broader sense, exoglucanases (EC 3.2.1.74 and EC 3.2.1.91) and β-glucosidases (EC 3.2.1.21) are also classified as cellulases [4,5]. In the modern world, cellulase has many applications in industry because of the wide existence of cellulose, lichenin, and cereal beta-D-glucans [6]. Many different types of organisms can produce cellulases, for example bacteria, fungi, protozoa, and some animal species, including termites and crayfish, which produce their own cellulases and differ substantially from those of their indigenous microflora [7].

Cellulase plays a mainly catalytic role in the extracellular matrix where the enzymatic reaction takes place [8,9]. Therefore, cellulases are produced from cells through a certain pathway, which may not be limited to the secretion of a single enzyme, but a group of proteins. For example the secretion of proteins in filamentous fungi may reach up to 20 g/L of extracellular medium [10] and the secretion of alkaline extracellular protease by *Yarrowia lipolytica* reaches 1 g/L [11].

A more complete knowledge of the cellulase secretory pathway will not only help us to understand this theoretical topic, but will also help the selection of organisms which most efficiently secrete cellulases, selection of cellulases which already exist in the extracellular matrix, and selection of organisms whose secretory pathways have less frequent mutations, and so on. This is important because cellulose is the most abundant component of plant biomass and has wide industrial applications, with a very promising prospective in the biofuel industry. Indeed, the conversion from biomass to biofuel can be divided into pretreatment, hydrolysis, fermentation, and distillation/evaporation [12], with cellulases involved in the whole process of hydrolysis.

A typical secretory pathway in a cell is generally composed of at least two components, endoplasmic reticulum and Golgi apparatus, and a typical cell generally has two endomembrane systems, one for incoming traffic and the other for outgoing traffic [13]. A protein generally undergoes the following process to be ready to move out of a cell: protein biosynthesis, translocation to endoplasmic reticulum, attachment of N-glycan, glycoprotein folding, N- and O-glycosylation, transportation to Golgi apparatus, protein sorting and formation of secretory vesicles, vesicle budding, transport, and vesicle fusion with the plasma membrane [13,14]. Those components form the general concept that eukaryotic cells use for the endoplasmic reticulum-to-Golgi membrane secretory pathway.

Various secretory cells exist, which include endocrine cells, exocrine cells, and immune cells [15-17]. For instance, in response to ultraviolet exposure, melanocytes synthesize melanin to form melanosomes and are then transferred to keratinocytes, which is considered to be a specialized type of secretion [18]. However, it is not yet known whether the cells that secrete cellulases belong to such specific secretory cells. Thus, there are a series of questions relating to the secretory pathway of cellulases that need answers by reviewing the literature, including: 1) Can we classify the cells that produce cellulases as secretory cells? 2) Where are the subcellular locations for cellulases? 3) Does a cellulase use the endoplasmic reticulum-to-Golgi membrane pathway for secretion? 4) Is a cellulase processed within the Golgi apparatus soluble? 5) Does a cellulase adopt a different secretory pathway from the common secretory pathway? 6) What are the special characteristics of cellulases for their secretion? Thereafter, we also hope to use the reviewed knowledge to examine cellulases documented in UniProt Knowledgebase (UniProtKB) [5], which was released on 24 July 2013 and included 4,101 cellulases with accession numbers.

In this context, it is necessary to address the secretary pathway of cellulase in this mini-review. With rapid advances in research facilities and technologies, the focus of research shifts rapidly across different levels. For example current research is heavily based on the genetic level, which would have been impossible several decades ago. A balanced review will not only address the results obtained from modern techniques but will also uncover the results obtained from earlier techniques, and play a complementary role to the understanding of the given problems.

## Location of cellulases in different organisms

### Subcellular locations in different organisms

In general, microorganisms cannot be considered as specialized secretory cells, such as the cells that secrete insulin, sweat, and so on, because secretion of proteins only accounts for a fraction of activities of microorganisms. However, the secretion of proteins in microorganisms still requires a series of operations between various subcellular locations, from synthesizing proteins to transporting them into the extracellular matrix.

A eukaryotic cell includes the following 21 subcellular locations: acrosome, cell wall, centriole, chloroplast, cyanelle, cytoplasm, cytoskeleton, endoplasmic reticulum, endosome, Golgi apparatus, hydrogenosome, lysosome, melanosome, microsome, mitochondrion, nucleus, peroxisome, plasma membrane, spindle pole body, synapse, and vacuole [19]. A human cell includes 12 subcellular locations: centriole, cytoplasm, cytoskeleton, endosomal, endoplasmic reticulum, Golgi apparatus, lysosome, mitochondria, nucleus, peroxisome, plasma membrane, and synapse. A plant cell includes ten subcellular locations: cell wall, chloroplast, cytoplasm, endoplasmic reticulum, mitochondria, nucleus, peroxisome, plasma membrane, plastid, and vacuole. A Gram-positive bacterium includes four subcellular locations: cell wall, cytoplasm, periplasm, and plasma membrane; and a Gram-negative bacterium includes seven subcellular locations: cytoplasm, fimbrium, flagellum, inner membrane, nucleoid, outer membrane, and periplasm [20].

A protein’s subcellular location can lead its specific function and critically influences cell functionality. For example the redox potential, which is maintained by enzymes such as oxidase and endothelial nitric oxide synthase [21], in a eukaryotic cell is proposed from most oxidizing to most reductive, as follows: mitochondrion > nucleus > cytoplasm > endoplasmic reticulum > extracellular [22]. Needless to say, the difference in redox potential has a direct impact on the cellular signaling system. On the other hand, an enzyme that has several subcellular locations could have different concentrations in order to function differentially.

### Subcellular locations of cellulases in different organisms

The subcellular locations of cellulases are a topic that has been studied for several decades, with different techniques available at given times. As early as the 1970s, the subcellular locations of buffer-soluble cellulase and buffer-insoluble cellulase from auxin-treated peas were studied [23], and the authors found that buffer-soluble cellulase was localized at the inner surface of the cell wall while buffer-insoluble cellulase was localized in the endoplasmic reticulum. This finding partially answered the question of whether cellulase is soluble, which is also confirmed by the fact that five types of secretory pathways in Gram-negative bacteria address soluble proteins [24]. Later on, the location of cellulase was suggested to be in cytoplasmic vesicles with 150 nm diameters by isolation of cellulase-containing membranes of *Achlya ambisexualis Raper*, and it was also found that IDPase, ATPase, UDPG transferase, and sedimentable carbohydrate were located in similar places [25].

Studies on fungi have demonstrated that the Golgi apparatus in fungi cells do not have a stacked appearance and some fungi have many individual endoplasmic reticulum-associated saccules [25-27]. Cellulase was found to be located in the vesicles that derived from endoplasmic reticulum, had ribosomes, and attached to the outside surface of the membrane [28].

β-glucosidases (EC 3.2.1.21) are grouped according to location, including intracellular, cell wall-associated, and extracellular [29]. For example β-1,4,-D-endoglucanase was found to be located on the outside surface of *Prevotella ruminicola*[30], which is a starch-degrading bacterium but utilizes water-soluble cellodextrins [31], and of which some strains have considerable carboxymethyl cellulase (CMCase) activity [32-35].

Enzymological studies have shown that bacterium has both extracellular and cell-bound endoglucanase activities, of which up to 80% were found in the extracellular fluid in the stationary-phase of cellulose-grown cultures. In *Fibrobacter succinogenes* subspecies *succinogenes* S85, a small part of endoglucanase was found in the periplasmic fraction while a large part was found in the cytoplasmic and membrane fractions [36]. Groleau *et al*. demonstrated that the majority of the cell-free cellulase was associated with sedimentable membrane fragments, and the rest was obtained from a fraction of low-molecular-mass that was approximately 45 kDa and nonsedimentable protein aggregates that were larger than 4 × 10^3^ kDa [37-39]. Also, three separate endoglucanases designated EG1, EG2 [40], and EG3 [41], an extracellular Cl-stimulated cellobiosidase [42], and a periplasmic cellodextrinase [43,44] were purified and characterized. The cellobiosidases were found to associate with actively growing cells in culture [44].

### Subcellular locations of cellulase described in UniProt Knowledgebase (UniProtKB)

Of 4,101 cellulases including 741 fragments from UniProtKB, 121 cellulases are evidenced at protein level, 306 cellulases are evidenced at transcript level, 217 cellulases are inferred from homology, and 3,457 cellulases are predicted. However, only 85 cellulases are documented with their subcellular locations. In Table 1, at protein level, 75% (24/32) of cellulases are annotated as a secreted form, that is, they were found in the extracellular matrix. At transcript level, 30% (3/10) of cellulases are annotated with their location in the nucleus, that is, these cellulases should be in eukaryotic cells. Cellulases inferred from homology resulted in 79.49% (31/39) in secreted form, that is, secreted cellulases are highly homologous rather than heterologous. In Table 2, 86.27% (44/51) of cellulases are annotated as secreted in eukaryotic cells, and this percentage is higher than those in Gram-negative and Gram-positive bacteria. This may imply that the secretory pathway in eukaryotic cells is more efficient than in Gram-negative and Gram-positive bacteria. It is not necessary that cellulases documented in UniProtKB are located in a single subcellular location, for example a cellulase from *Streptomyces reticuli* [UniProt:Q05156] was found to exist in both mycelium-associated and extracellular forms.

**Table 1:** Subcellular locations of cellulases in UniProtKB according to the sequence status

**Sequence status**	**Cell membrane**	**Cytoplasm**	**Nucleus**	**Periplasm**	**Secreted**	**Total**
Evidence at protein level	5 (15.63%)	1 (3.13%)	0	2 (6.25%)	24 (75%)	32
Evidence at transcript level	0	0	3 (30%)	0	7 (70%)	10
Inferred from homology	2 (5.13%)	6 (15.38%)	0	0	31 (79.49%)	39
Predicted	0	1 (25%)	2 (50%)	0	1 (25%)	4
Total	7 (8.24%)	8 (9.41%)	5 (5.88%)	2 (2.35%)	66 (74.12%)	86

**Table 2:** Subcellular locations of cellulases in UniProtKB according to cell types

**Cell**	**Cell membrane**	**Cytoplasm**	**Nucleus**	**Periplasm**	**Secreted**	**Total**
Eukaryota	2 (3.92%)	0	5 (9.8%)	0	44 (86.27%)	51
Gram-negative bacteria	3 (15%)	3 (15%)	0	2 (10%)	11 (55%)	20
Gram-positive bacteria	2 (15.38%)	4 (30.77%)	0	0	8 (61.54%)	13
Undetermined bacterium	0	1 (100%)	0	0	0	1
Total	7 (8.24%)	8 (9.41%)	5 (5.88%)	2 (2.35%)	63 (74.12%)	85

## Secretory pathways in different organisms

### Secretory pathways in general

In the late 1980s, the term autotransporter was coined for secreted proteins as a type of secretory pathway in Gram-negative bacteria, because Gram-negative bacteria have two asymmetric biological membranes while Gram-positive bacteria have only one. To date, seven types of secretory pathways have been defined for secreting soluble proteins in Gram-negative bacteria [45,46]. Type I secretory pathway includes an oligomeric complex composed of an inner membrane ATP-binding cassette exporter, a membrane fusion protein, and an outer membrane homologue [47,48]. Type II secretory pathway, which has an alternative name as the general secretory pathway, presents a two-step process: 1) proteins are moved across the inner membrane through the Sec system; and 2) proteins are moved across the outer membrane [49,50]. Type III secretory pathway is a highly regulated channel through both the inner and outer membranes forming a needle-like structure [51-53]. Type IV secretory pathway involves the conjugative transfer of DNA as well as nucleoprotein complexes, and is further divided into type IVa and IVb according to sequence homology [53-55]. Type V secretory pathway has the simplest secretion apparatus and represents a large family of protein-translocating outer membrane porins [56]. Type VI secretory pathway is a newly discovered pathway [57,58], which spreads in Gram-negative bacteria, and plant and human pathogens as well [59-61]. For *Mycobacterium*, there is the type VII secretory pathway, which also exists in Gram-positive bacteria but to a far less extent [62-64]. However, type VII is not related to cellulase according to the current knowledge.

Gram-positive bacteria have fewer subcellular locations and simpler membranes than those in Gram-negative bacteria, and do not have a dedicated apparatus for folding secreted polypeptides. However, this by no means implies that Gram-positive bacteria have fewer secretory pathways. In fact, several secretory pathways are found in Gram-positive bacteria: secretion (Sec), twin-arginine translocation (Tat), flagella export apparatus (FEA), fimbrilin-protein exporter (FPE), hole forming (holin), and the WXG100 secretion system (Wss) [65]. However, the Sec secretory pathway is the most relevant to protein secretion because a Sec-type pathway is used in an important human pathogen, *Streptococcus pyogenes*, which proceeds through a single microdomain [66], and the involvement of Sec-type pathways [67-69] is more likely to be SecA because secretion of cytotoxins can be inhibited by the SecA inhibitor sodium azide in *Bacillus cereus*.

In mammalian cells, the vesicles that contain synthesized proteins travel along microtubules from the rough endoplasmic reticulum towards the cis-Golgi or an endoplasmic reticulum-to-Golgi intermediate compartment (ERGIC). It is noted that a number of proteins never enter into this pathway, and these proteins are generally considered to be involved in cell survival, immune surveillance, and tissue organization with fundamental importance. Therefore, it is proposed that at least the secretion of these proteins can be classified according to whether the proteins go through a non-vesicular secretory pathway or a vesicular secretory pathway. For the non-vesicular secretory pathway, two types have been defined: type I is a self-sustained protein translocation across plasma membranes, and type II is an ATP-binding cassette transporter-based secretion. For the vesicular secretory pathway, two types have also been defined: type III is an autophagy-based secretion, and type IV comprises proteins that bypass the Golgi apparatus to transport to the plasma membrane. However, types I, II, and III are involved in the secretion of cytoplasmic proteins while type IV consists of integral membrane proteins [70] which usually have distinct regions of hydrophobicity [71-73].

A typical secretory pathway in a eukaryotic cell begins with budding from the endoplasmic reticulum to forming the coat protein complex II (COPII) vesicles either fully or partially uncoated [74]. In yeast, protein secretion has been well-studied because yeast has cell wall synthesizing enzymes [75,76]. It has been suggested that the essential functions through the endoplasmic reticulum membrane, primary glycosylation, folding and quality control, and vesicle-mediated secretion are similar from yeasts to higher eukaryotes. However, recent research has indicated that significant functional differences exist between yeasts and mammalian cells [77].

The secretory pathway in Archaea has been mainly studied using genomic sequencing data, and compared against the secretory pathways in bacteria and Eucarya [78,79]. It was found that the secretory pathway in Archaea is similar to the Sec system in bacteria and Eucarya [80] because most Archaea have a homologue of CsaA, a protein involved in protein targeting in *Bacillus subtilis*[81,82], although they have a lipid monolayer instead of a phospholipid bilayer.

Table 3 summarizes the general secretory pathways in different organisms. Although secretory pathways are termed and classified with a small number of apparatuses in cells, numerous different types of proteins have been identified as major components for the construction of secretory pathways, including membrane traffic and protein secretion [14]. The secretion of proteins is also called protein production. However, the secretory pathway is not limited to secreted proteins produced by the organism itself, but also involves secreted foreign substances, for example drugs and their metabolites [83], and secreted cytotoxic substances, such as orphan granzymes [84].

**Table 3:** List of general secretory pathways in different organisms

**Organism**	**Secretory pathway**	**Description**
Gram-negative bacteria	Type I secretory pathway	An oligomeric complex composed of an inner membrane ATP-binding cassette exporter, a membrane fusion protein, and an outer membrane homologue.
	Type II secretory pathway, also known as the general secretory pathway	A two-step process: 1) proteins are moved across the inner membrane through the Sec system, and 2) proteins are moved across the outer membrane.
	Type III secretory pathway	A highly regulated channel through both the inner and outer membranes forming a needle-like structure.
	Type IV secretory pathway	Involves conjugative transfer of DNA and nucleoprotein complexes.
	Type V secretory pathway	A large family of protein-translocating outer membrane porins.
	Type VI secretory pathway	Forms a transenvelope apparatus. It also exists in plant, animal, and human pathogens, and environmental strains.
	Type VII secretory pathway	Exists mainly in *Mycobacterium* and Gram-positive bacteria to a small degree.
Gram-positive bacteria	Sec-type pathways	Involves Sec-type signal peptides.
Mammalian cells	Non-vesicular secretory pathway	Type I is a self-sustained protein translocation across plasma membranes.
		Type II is an ATP-binding cassette transporter-based secretion.
	Vesicular secretory pathway	Type III is an autophagy-based secretion.
		Type IV comprises the proteins that bypass the Golgi apparatus to transport to the plasma membrane.
Eukaryotic cells	Budding from endoplasmic reticulum to form the coat protein complex II (COPII) vesicles	Essential processes are similar from yeasts to higher eukaryotes.
Archaea	Similar to Sec-type pathways	Most Archaea have a homologue of CsaA.

### Secretory pathways of cellulase

A single organism is not limited to secretion of a single type of cellulase but several different types, which work in a synergistic manner. This is similar to the secretion of hemicellulase, for example aerobic fungi *Trichoderma reesei* and *Aspergillus niger* secrete 8 and 12 hemicellulases in high concentrations, respectively [85]. Fungi, such as *T. reesei* and *A. niger*, produce large amounts of extracellular cellulolytic enzymes, whereas some strains, including bacteria mainly from the class Clostridia, such as *Clostridium cellulovorans*[86-88] and *Clostridium thermocellum*[89,90], a few anaerobic fungi [91,92], and *Eisenia fetida*[93], mostly produce cellulolytic enzymes in a multienzyme complex called cellulosome, which is associated with the degrading cell wall [94,95].

Secreted proteins can include homologous and heterologous proteins, for example a typical cellulosome can be composed of 20 or more different cellulolytic/hemicellulolytic enzymes in anaerobic bacteria, while in anaerobic fungi, for example *Neocallimastix frontalis* and *Piromyces*, a cellulosome-type complex includes at least six or ten polypeptides [92,95,96]. Accordingly, an 18-subunit protein complex has been engineered to multienzyme structures called rosettasomes [97]. The modeling of cellulosome self-assembly showed that the shape and modularity were the dominant factors influencing the cellulosome assembly process [98].

### Secretory pathways of cellulase described in UniProtKB

**Figure 1:**
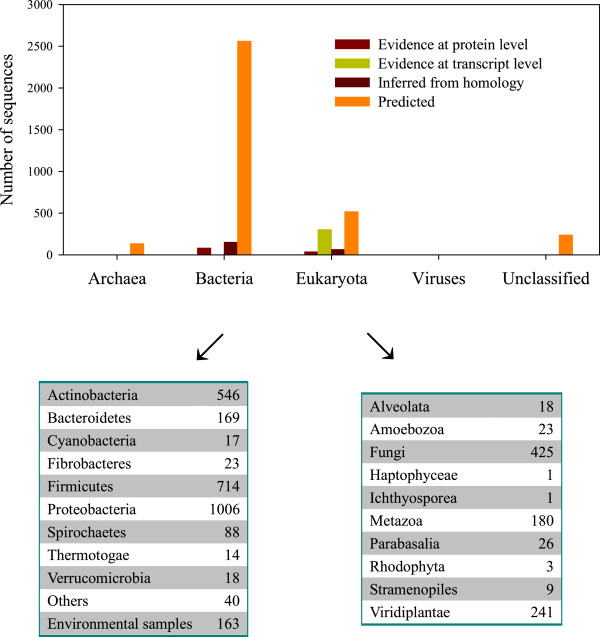
Summary data of organisms secreting cellulases documented in UniProtKB.

Of the 4,101 cellulases in UniProtKB, 133 cellulases come from Archaea, 2,799 from bacteria, 928 from Eukaryota, and only two from viruses, and the remaining 239 are unclassified (upper panel of Figure 1). Therefore, a vast majority of cellulases in UniProtKB come from bacteria and Eukaryota, of which further classification is listed in the lower panel of Figure 1. Since the secretory pathways in bacteria are defined according to Gram-negative and Gram-positive, excluding undetermined bacteria, there are 1,210 Gram-positive bacteria and 1,365 Gram-negative bacteria in UniProtKB. Therefore, when addressing cellulases from UniProtKB, it is likely that a cellulase will follow one of seven secretory pathways defined in Gram-negative bacteria. Indeed, cellulase [UniProt:Q38890] is annotated as a single-pass type II membrane protein, but from a plant cell. The type V secretory pathway is unlikely to exist in bacteria secreting cellulases, because type V operates on the autotransporter. A search for autotransporter proteins in UniProtKB does not reveal any cellulases belonging to the autotransporter; however, *Yersinia pestis* does have nucleotide sequences checkable for autotransporter search. Therefore, 124 cellulases from *Y. pestis* may be considered as candidates for the type V secretory pathway in Gram-negative bacteria.

## Secretory mechanisms

### Secretory mechanisms in general

It is estimated that a third of proteins synthesized within a cell come from the endoplasmic reticulum [14]. The secretion is triggered by signal peptide-dependent protein translocation into the lumen of the endoplasmic reticulum, followed by vesicular transport of secretory cargo to the Golgi apparatus membranes, and thereafter to the cell surface. In addition, the proteins known as tethering factors are needed to build an initial connection between vesicles and the destination membrane [74,99]. In this view, for many proteins that are secreted into the extracellular matrix, their subcellular locations are dynamically regulated by various mechanisms. For example, although the secretory pathway in Gram-negative bacteria is related to two membranes, seven types operate in different mechanisms.

For the type I secretory pathway, the mechanism shown in *Escherichia coli* with HlyA toxin is signal sequence-independent without free periplasmic intermediate [45,100]. The secretion is undertaken by a translocator spanning the cell envelope with three proteins: 1) a specific outer membrane protein, 2) a cytoplasmic membrane protein, ATP-binding cassette, and 3) a cytoplasmic membrane protein, membrane fusion/adaptor protein. The secreted proteins do not require a cleaved C-terminal signal, because this signal sufficiently recognizes the ATP-binding cassette protein leading to the assembly of the functional transenvelope complex. [101]. It is estimated that the transport of polypeptides up to 900 kDa across the cell envelope takes a few seconds.

For the type II secretory pathway, the mechanism operates at two different locations, as shown in studies on the pullulanase enzyme from *Klebsiella oxytoca*. The first mechanism involves the Sec system to transport proteins across the inner membrane, while the second involves the transport of effector molecules across the outer membrane, which works for a specific secretion apparatus, secreton, composed of up to 16 different gene products and at some stage spanning the entire cell envelope [49,50], and general secretion proteins (GspD, GspE, and GspF) help the second mechanism [102]. When examining the type II secretory pathway, the Sec system should be considered, which works for the first part of the working mechanism of the type II secretory pathway. Proteins with N-terminal signal peptides are understood to be secreted by the Sec-dependent pathway [49]. SecA is an ATP-dependent motor protein, but is associated with the SecYEG complex on the cytoplasmic side and promotes the access of the chaperone-assisted substrate proteins to the inner membrane SecYEG complex [103]. SecB, a secretion-dedicated chaperone, can recognize cytosolic protein precursors [104,105]. Both SecE and SecG are auxiliary molecules for the translocase activity, while SecY is absolutely necessary [106]. SecYEG is a translocase complex in the cytoplasmic or inner membrane and uses a substrate that can be targeted by a G protein precursor [107-110]. Additional observations of type II have been found in *Erwinia carotovora*, *Erwinia chrysanthemi* (Echr), *K. oxytoca*, *Pseudomonas aeruginosa*, *Xanthomonas campestris*, and *Aeromonas hydrophila*[45,111].

For the type III secretory pathway, the mechanism is well-characterized in *Salmonella* and *Yersinia*. It has a complex apparatus, which is also named injectisome or molecular needle, because assembled proteins form a highly regulated channel through both the inner and outer membranes in a needle-like structure. This apparatus secretes proteins not only into the extracellular milieu but also directly into a target eukaryotic cell. In fact, many Gram-negative plant and animal pathogenic bacteria use this system as a molecular syringe to inject effector proteins directly into the host cell. The regulation of this secretory pathway is mediated by a bacterial translocon, and there are several putative secretion translocon proteins that have been identified with structural similarity [51].

For the type IV secretory pathway, the mechanism seems to be more related to clinical settings, where bacterial conjugation is problematic because it leads to a rapid dissemination of antibiotic resistance genes and other virulence traits among bacterial populations. This pathway delivers effector molecules, including DNA and protein substrates such as the pertussis toxin, as well as monomeric proteins such as RecA, to influence eukaryotic target cells during infection. Thus it is also known as a macromolecular transfer system and found in several pathogens of plants and mammals, including *Agrobacterium tumefaciens*, *Bordetella pertussis*, *Helicobacter pylori*, and *Legionella pneumophila*. Two subclasses have been defined based on sequence homology [54]: 1) type IVa refers to the machinery assembled from VirB homologues of *A. tumefaciens*, and 2) type IVb refers to the functional secretion system assembled from Tra homologues of the Incl ColIb-P9 plasmid of *Shigella flexneri*[55].

For the type V secretory pathway, the mechanism was first described for the IgA1 protease produced by *Neisseria gonorrhoeae*[112]. This pathway has the simplest secretion apparatus, is composed of the largest family of protein-translocating outer membrane porins in Gram-negative bacteria [56], and requires the protein precursors to have three functional domains: 1) the N-terminal leader initiating the inner membrane transport of the precursor, 2) the mature part of the protein forming the extracellular functional domain, and 3) a C-terminal helper domain that is essential for extracellular secretion.

For the type VI secretory pathway, the mechanism seems to be adapted by individual bacterial species to interact with other prokaryotes, eukaryotes, or both [113], and the structure forms a transenvelope apparatus [114].

The aforementioned mechanisms are related to only how a protein is secreted, while the mechanisms that regulate each secretory pathway are far more complicated. This is not only because current research focuses on the genes, which produce protein regulating secretions, but also because those regulating proteins are subject to the regulation of environments. With regard to the regulation mechanism at a genetic level, the type II secretory pathway in *E. carotovora* subspecies *carotovora* is encoded by the out cluster, which has 15 out genes termed outB-0 and outs. This out cluster has been sequenced and the resulting secretion defective mutants (Out-) have been isolated [115]. There is little doubt that the list of genes involved in regulating the secretory pathway is increasing. For example Mdr49 is a functional homolog of Ste6 and mediates the ATP-binding cassette transporter in *D. melanogaster*[116]. With regard to the regulation mechanism at environmental level, Ca^2+^ plays a role in the secretory pathway [117]. For instance, there is a Ca^2+^-dependent growth defect induced by the *PMR1*-disrupted mutant [118-120]. There are also many environmental factors that can affect the functionality of the secretory pathway, such as pH.

### Secretory mechanisms of cellulase

A general consideration on the secretion of cellulases suggests that there are three different mechanisms based on their subcellular locations: 1) a specific secretory pathway independent of cellulose, 2) a secretory pathway which is induced by cellulose, and 3) a generalized blebbing process that occurs irrespective of the carbon source [36]. The cellulase secretion needs to be induced, and this induction can include the generation of new proteins for constructing secretory pathways. Taking an example of induction of cellulases and hemicellulases by D-xylose, Ferreira de Oliveira *et al*. found that 282 proteins were induced by D-xylose and 161 proteins were induced by sorbitol, while another 638 proteins were presented under both conditions in mycelia from *A. niger*; of which 254 proteins were predicted to relate to the secretory pathway [14]. The cellulose itself can also trigger the secretion of endoglucanases as shown in an early study by McGavin *et al*. [36]. Extracellular concentration of chlorine can also stimulate the secretion of cellobiosidase [43]. It has been shown that the secretion system for cellulase in *E. carotovora* subspecies *carotovora* (Ecc) belongs to type II of Gram-negative bacterium, and is highly homologous in a wide range of bacteria [45,111,121]. As early as the 1980s, the issue of whether synthesis of endoglucanase activity was regulated by a carbon source was studied in *C. thermocellum*[122-126].

With regard to anterograde and retrograde transport, the secretory pathway was depicted with the following steps: 1) N-glycan biosynthesis and transfer to asparagine in normally glycosylated target proteins, 2) endoplasmic reticulum-to-Golgi network anterograde and retrograde transport, 3) Rab GTPases and interacting factors mediate this process, 4) process related to microtubules, and 5) the endoplasmic reticulum-associated degradation pathway as the early checkpoint [14]. Accordingly, the secretion of cellulase is a forward (anterograde) process rather than a backward (retrograde) process. In such a case, the secretory pathway should be regulated by Rab GTPase, which is a group of proteins from the Ras superfamily of monomeric guanosine triphosphatases and includes over 150 structurally closely related members [13]. However, the Ras family is mainly found in humans. For example Rab27, an effector in the regulation of secretory pathways, is not found in yeasts and plants [127]. The consideration given to Rab27 is partially due to the fact that the cells that secrete cellulase are not professional secretory cells, while Rab27 is involved in the secretion of exosomes in non-secretory cells [128], which again supports the idea that the cells secreting cellulase are not specific secretory cells. On the one hand, endosomes deal with incoming traffic, that is, to endocytose proteins, sort, recycle, and process degradation of internalized cargos. However, on the other hand, the exosomes in most cell types are derived from intraluminal vesicles of multivesicular endosomes, form small membrane vesicles, and contribute to intercellular communications [129].

Mutations can certainly change cellulase production, for instance, when grown on crystalline cellulose, a significantly high amount of cellulase can be synthesized and secreted by a mutant fungus of *T. reesei* QM6a (mutant RUT-C30). On the contrary, the cellulase activity decreases at late stages of the growth of wild-type cells because they can only secrete a small amount of cellulase [28].

### Secretory mechanism described in UniProtKB

**Figure 2:**
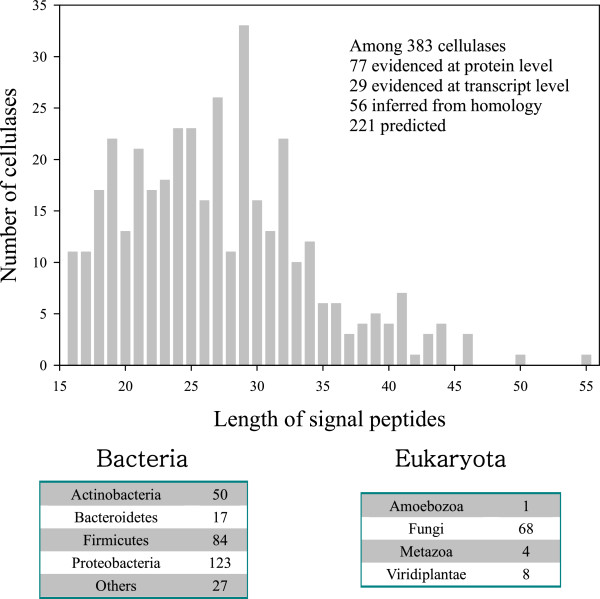
Length distribution of signal peptides of cellulases obtained from UniProtKB with their secreting organisms.

Up until now, the literature review reveals that the secretory pathway for most cellulases is most likely to be the type II secretory pathway, for which the signal region in cellulase plays a role to initiate the secretion of cellulase into the extracellular matrix. Therefore, it is not unnecessary to examine the signal region of cellulases in UniProtKB in order to gain a general overview of this issue. Of 4,101 cellulases documented in UniProtKB, 386 cellulases have a signal region in their sequence. With exception of three cellulases, the length of signal peptides is 27 ± 7 (mean ± SD) amino acids ranging from 16 to 65 amino acids (Figure 2). It is said that the yeast secretary signal is an alpha-factor fragment [130] and can be predicted using the PSORT program [131]. In UniProtKB there are ten yeasts, whose signal regions are considered to be similar to the alpha-factor fragment.

However, 3,713 cellulases are yet to have the signal region in their sequence annotated, which could be due to technical reasons or could otherwise suggest that the secretion of these cellulases go through other secretory pathways. This may be possible because the five types of secretory pathway, whose mechanisms are clearly illustrated, are referenced to Gram-negative bacteria. Indeed, the mechanism that regulates the secretion of non-vesicular proteins using the ATP-binding cassette transporter is important for biotechnology because this pathway belongs to the type I pathway and seems to translocate lipidated peptides and proteins across the plasma membrane of eukaryotic cells. For example the yeast alpha-factor pheromone, a farnesylated peptide, is transported in this manner by the ATP-binding cassette transporter Ste6 [132,133], and the farnesylated M-factor of *Schizosaccharomyces pombe* is also transported by the ATP-binding cassette transporter Mam1 [134]. Yet, acylated proteins, such as the hydrophilic acylated surface protein B in *Leishmania* species [135-137] and *Plasmodium falciparum* Ca^2+^-dependent protein kinase 1 in *P. falciparum*, are exported to the parasitophorous vacuole in parasites [138]. These findings suggest that various lipidated peptides and proteins are secreted by ATP-binding cassette transporters in eukaryotic organisms [70].

## Conclusions: implications and knowledge gaps

Although the secretory pathway is a popular research topic, the present review indicates that this topic has yet to draw sufficient attention in cellulase research. However, the literature review suggests that cellulases share their secretory pathway with other secreted proteins rather than having a specific secretory pathway. Therefore, the knowledge obtained from studies on secretory pathways generally benefits our understanding on the secretory pathway of cellulase.

Of the 4,101 cellulases in UniProtKB [5], a few were annotated with enzymatic activity of cellulases under different circumstances. For example the optimal pH for a cellulase reaction is 5.26 ± 1.4 (mean ± SD, n = 8) for the cellulases obtained from Eukaryota and 6.39 ± 1.49 (mean ± SD, n = 11) for the cellulases obtained from bacteria, while the optimal temperature for a cellulase reaction is 53 ± 11.58°C for the cellulases obtained from Eukaryota and 53.78 ± 17.28°C for the cellulases obtained from bacteria.

The literature review has revealed several gaps in knowledge with regard to the secretory pathway of cellulases, including the following:

1) The subcellular location of cellulases is still not clear in some organisms, such as protozoa, although some studies have been undertaken [139]. At present, this shortage could be compensated by the fact that protozoa are a diverse group of unicellular eukaryotic organisms [140]. Therefore, our understanding on the subcellular locations of cellulases in eukaryotes could be applied to protozoa.

2) As cellulase needs to be coated with a vesicle, it is possible that cellulases could be involved in exosomes as well as endosomes, whose function deals with incoming traffic. Is it possible that cellulases can be transferred back into cells, considering experimental observations indicate a decline in enzymatic activity with time?

3) It is widely understood that many proteins need to be folded in order to transport through the membrane (for example fibroblast growth factor 2 has to be fully folded to pass through the plasma membrane [141-143]). However, it is not clear whether a cellulase needs to be folded in order to transport through the secretory pathway.

4) There is a shortage of studies on the mechanisms regulating the secretory pathway of cellulase at both genetic and environmental levels. For instance, it is not clear whether inhibitors could help to stop the secretion of other proteins that share the same secretory pathway with cellulase. It is also not clear whether osmolality influences the secretory pathway of cellulase, since it would be expected that the concentrations of substrate and product change continuously during fermentation leading the osmolality to change together with pH, temperature, pressure, and so on.

5) A considerable number of studies have been undertaken to mutate genes in order to enhance the activity of cellulase [144,145]. However, the literature review could suggest a way to increase the secretion of cellulase through the mutations in the secretory pathway of cellulase. For example a mutation in the *PMR1* gene resulted in a 5- to 50-fold increase in the secretion of bovine growth hormone, prochymosin [121,146].

6) As most secretory and cell-surface proteins contain disulfide bonds [147,148], it is not clear whether cellulases should undergo the process to have disulfide bonds during transportation, while the cellulases that do have disulfide bonds are among the 4,101 cellulases from UniProtKB, such as the cellulase [UniProt:P07103].

## Abbreviations

CMCase: Carboxymethyl cellulase; COPII: Coat protein complex II; ERGIC: Endoplasmic reticulum-to-Golgi intermediate compartment; FEA: Flagella export apparatus; FPE: Fimbrilin-protein exporter; GSP: General secretory pathway; holin: Hole forming; Sec: Secretion; Tat: Twin-arginine translocation; UniProtKB: UniProt Knowledgebase; Wss: WXG100 secretion system.

## Competing interests

The authors declare that they have no competing interests.

## Authors’ contributions

GW conceived the idea and wrote the first draft of the manuscript. SMY and GW contributed to manuscript revision and approved the final version.

## Authors’ information

GW received his MD from Tianjin Medical University, Tianjin, China (1984), PhD from Russian Medical University, Moscow, Russia (1992), did postdoctoral research at University of Udine, Udine, Italy (1992 to 1993), Institute for Minamata Disease, Minamata, Japan (1993 to 1994), University of Udine, Udine, Italy (1994 to 1999), University of Marseilles, Marseilles, France (2000), and worked at Novartis Pharma AG, Basel, Switzerland (2001 to 2002). GW worked as a general manager at DreamSciTech Consulting company, Shenzhen, China (2002 to 2012), and since 2008, has worked as a guest professor at Guangxi Academy of Sciences, Nanning, China. GW has published over 170 research papers, including over 120 research papers in Science Citation Index (SCI) journals and six books in the USA and Germany.

SMY received her MD from Tianjin Medical University, Tianjin, China (1984), MS from Tianjin Medical University, Tianjin, China (1987), PhD from University of Siena, Siena, Italy (2000), and did postdoctoral research at University of Udine, Udine, Italy (1999 to 2001). SMY worked as a director at DreamSciTech Consulting company, Shenzhen, China (2002 to 2012), and since 2008, has been working as a professor at Guangxi Academy of Sciences, Nanning, China. SMY has published over 140 research papers including over 80 research papers in SCI journals and two books in the USA and Germany.
